# Temporomandibular Disorders and Oral Parafunctions: Mechanism, Diagnostics, and Therapy

**DOI:** 10.1155/2015/354759

**Published:** 2015-05-14

**Authors:** Klaus Boening, Mieszko Wieckiewicz, Anna Paradowska-Stolarz, Piotr Wiland, Yuh-Yuan Shiau

**Affiliations:** ^1^Department of Prosthetic Dentistry, Faculty of Medicine, Dresden University of Technology, 01307 Dresden, Germany; ^2^Department of Prosthetic Dentistry, Faculty of Dentistry, Wroclaw Medical University, 50425 Wroclaw, Poland; ^3^Department of Maxillofacial Orthopedics and Orthodontics, Faculty of Dentistry, Wroclaw Medical University, 50425 Wroclaw, Poland; ^4^Department and Clinic of Rheumatology and Internal Medicine, Faculty of Medicine, Wroclaw Medical University, 50556 Wroclaw, Poland; ^5^Department of Prosthetic Dentistry, School of Dentistry, National Taiwan University, Taipei City 100, Taiwan

Temporomandibular disorders (TMD) and oral parafunctions are very common problems in the modern society. TMD are a group of symptoms related to impaired function of the temporomandibular joints (TMJs) and associated muscles. The symptoms can include pain or tenderness of TMJs area, clicking or grating sounds in the TMJs, limited jaw movements, muscle pain, headache, tinnitus, impaired hearing, and earache. It had been proved that they are related to multiple causes, such as psychological, occlusal, and general health factors [1–3]. There is also evidence that TMD may be related to cervical spine disorders and its mobility [4, 5].

The paradigm shift and the growing awareness that diagnosis and treatment of TMD usually require a multidisciplinary approach were the goal intention to initiate a special issue on this topic. Interdisciplinary therapeutic strategies should focus not only on TMJs structures, but also on the surrounding tissues including especially neuromuscular system and last but not least the entire patient and his or her social environment [6]. Regarding the difficulties in diagnosis and multipronged treatment which is due to the symptom diversity and the complexity of associated problems, it was the editors' intention to condense the knowledge on temporomandibular disorders from different perspectives for the readers of this special issue.

In this special issue original and review articles related to TMD and oral parafunction topics are associated with multiple branches of medicine. The papers underline the multidisciplinary character of TMD to the readers. The aim of the issue was also to show novelties and advances in the treatment of TMD. A number of papers describe the pathogenesis of the disorders, as well as its epidemiology, stat-of-the-art diagnostics, and treatment methods.

The goal of the special issue was to familiarize the reader with multidimensional causes related to the specific disease process of TMD.
